# Performance evaluation of an automated image registration algorithm using an integrated kilovoltage imaging and guidance system

**DOI:** 10.1120/jacmp.v7i1.2199

**Published:** 2006-02-21

**Authors:** Timothy Fox, Calvin Huntzinger, Peter Johnstone, Tomi Ogunleye, Eric Elder

**Affiliations:** ^1^ Varian Medical Systems Palo Alto California U.S.A.; ^2^ Radiation Oncology Department Emory University School of Medicine Atlanta Georgia

**Keywords:** image‐guided radiation therapy, image registration, quality assurance

## Abstract

Image‐guided radiation therapy delivery may be used to assess the position of the tumor and anatomical structures within the body as opposed to relying on external marks. The purpose of this manuscript is to evaluate the performance of the image registration software for automatically detecting and repositioning a 3D offset of a phantom using a kilovoltage onboard imaging system. Verification tests were performed on both a geometric rigid phantom and an anthropomorphic head phantom containing a humanoid skeleton to assess the precision and accuracy of the automated positioning system. From the translation only studies, the average deviation between the detected and known offset was less than 0.75 mm for each of the three principal directions, and the shifts did not show any directional sensitivity. The results are given as the measurement with standard deviation in parentheses. The combined translations and rotations had the greatest average deviation in the lateral, longitudinal, and vertical directions. For all dimensions, the magnitude of the deviation does not appear to be correlated with the magnitude of the actual translation introduced. The On‐Board Imager™ (OBI) system has been successfully integrated into a feasible online radiotherapy treatment guidance procedure. Evaluation of each patient's resulting automatch should be performed by therapists before each treatment session for adequate clinical oversight.

PACS numbers: 87.53.‐j, 87.53.Xd, 87.57.Gg

## I. INTRODUCTION

Image‐guided radiation therapy delivery may be used to assess the position of the tumor and anatomical structures within the body as opposed to relying on external marks.^(^ ^1^ ^–^ ^4^ ^)^ Tumors or nearby critical structures may be displaced on a daily basis, which is referred to as interfraction motion. Electronic portal imaging devices (EPIDs)^(^ ^5^ ^–^ ^10^ ^)^ have been implemented to improve geometric accuracy of treatment delivery procedures. The standard technique used for interfraction motion management is the comparison of megavoltage (MV) X‐ray EPIs to kilovoltage (kV) simulation images. An EPI is acquired with an electronic imaging system on the MV treatment delivery system. Simulation films or digitally reconstructed radiographs (DRRs) are obtained as the reference image with kV imaging techniques. Determination of setup error is performed using these projection radiographs by measuring the positions of internal or external landmarks. The use of EPI is limited by the low contrast of soft tissue at MV energies; correction strategies are susceptible to additional uncertainty due to the differences in image quality and apparent object dimension between the prescription images acquired with kV X‐rays and the verification images acquired with MV X‐rays.^(^ ^11^ ^,^ ^12^ ^)^

An integrated imaging system has been developed to provide kV imaging of the anatomy to improve the accuracy of patient positioning for both interfraction and intrafraction motion management. This On‐Board Imager™ (OBI) system (Varian Medical Systems, Palo Alto, CA) consists of a kV X‐ray tube, a kV fluoroscopic imaging system, and an amorphous‐silicon flat panel digital imaging detector, which are attached to a medical LINAC. The purpose of this manuscript is to evaluate the performance of OBI software for automatically detecting and repositioning a 3D offset of a phantom. The elements of the OBI system, registration software, and phantom validation of automated image registration are reported. These results are critical for the application of this technology in routine clinical practice.

## II. METHODS AND MATERIALS

### A. Hardware system

**Figure 1:**
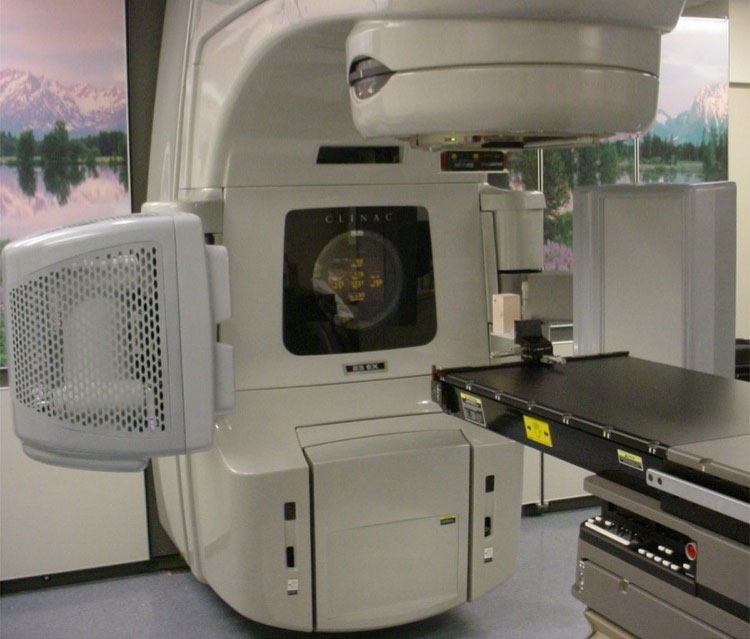
The OBI system. The kV source and imaging panel with our LINAC are shown with the robotic arms in the full extended position. The source and panel can be remotely retracted when treating the patient.

A Varian 23EX (Varian Medical Systems, Palo Alto, CA) LINAC forms the basis of the integrated imaging system. This accelerator is computer‐controlled and produces 6‐MV and 15‐MV photon beams. Field shaping is performed with a Millennium 120‐leaf multileaf collimator. The OBI system is composed of an X‐ray tube (model G242, 0.4‐mm and 0.8‐mm focal spots, 14° anode angle, 800 kJ/h, Varian Medical Systems, Inc., Salt Lake City, UT) and an amorphous‐silicon imaging panel (model PaxScan® 4030CB, Varian Medical Systems, Inc., Salt Lake City, UT) attached to the gantry of the LINAC with Exact™ robotic arms (Varian Medical Systems, Baden, Switzerland). Figure 1 shows the OBI system for our LINAC. The imaging panel has an active area of $300\times 400\;{\text{mm}}^{2}$ capable of operating in a fine resolution mode (2048×1536 pixels at 7.5 frames/s) or a standard resolution mode (1024×768 pixels at 15 frames/s). The fine resolution mode is used for high‐quality digital X‐ray radiographs with 0.195 mm per pixel resolution. The standard resolution mode is used for both single radiographic exposures and continuous digital fluoroscopy at 0.390 mm per pixel. Three motorized pivot points on the robotic arms allow OBI to be remotely extended or retracted from the control console during patient treatments. The focal spots of the X‐ray tube and the MV source are 90° apart and share a common isocenter. The kV X‐ray source can be located between 80 cm and 100 cm from the machine isocenter, and the MV source is fixed at 100 cm from the machine isocenter. A kV source collimator is used for symmetrical and asymmetrical fields with the blade positions defined by the user. With the X‐ray collimator fully open and the imager at the maximum distance from the source, the maximum field size at isocenter is $50\times 50\;{\text{cm}}^{2}$ and $91\times 91\;{\text{cm}}^{2}$ at the imaging detector.

### B. Software and automated image registration

The OBI system is controlled by a separate console at the treatment control station. The OBI control console mechanically enables and moves the imaging arms from outside the treatment delivery room. A foot switch is used to initiate the kV beam during imaging of the patient. An OBI software system supports the verification process for patient localization and the maintenance of the OBI system. Software version 1.1 was used for measurements reported in this paper. A quartet of images—two reference images and two newly acquired images—is used for the imaging matching, which can be performed manually or automatically with the system. The manual match allows the user to translate the images in the couch lateral, longitudinal, and vertical directions as well as the couch rotation (yaw) direction. The image match verification is reviewed using gray scale blending, color blending, split windows, and contour overlay segmented from the CT dataset. The OBI software incorporates an automated iterative 2D‐2D matching algorithm based on mutual information (MI) and an iterative optimizer based on gradient descent. It will be briefly described here in the context of its implementation with OBI.

The use of MI for medical image registration applications was independently introduced in 1995 by both Viola and Wells^(13)^ and Collignon et al.^(14)^ For two images, the mutual information is computed from the joint probability distribution of the images’ pixel intensity values. When two images are aligned, the joint probability distribution is a sharp peak resulting in a high MI value.

The clinical purpose of the automated matching procedure is to determine the position of the patient's anatomy in the field of view of the OBI imaging system compared to its position in DRRs derived from the treatment‐planning CT scan. The automated MI algorithm searches for a transformation between the newly acquired OBI kV images and the treatment planning‐derived DRR images at which identical anatomical landmarks of both images are most closely overlapping. A general optimizer seeks the global maximum of a cost value (provided by the cost function) by iteratively modifying the search parameters according to the optimization scheme. The cost function in the automated OBI software uses an image similarity measure that provides an assessment of the registration between reference DRRs and newly acquired kV OBI images. The similarity measure based on MI is used to compare the pixel intensities in the OBI image, $I_{\text{OBI}}(i,\;j)$, with the pixel intensities in the DRR, $I_{\text{DRR}}(i,\;j)$, where (*i*, *j*) defines the position of a pixel to be in column *i* and row *j* of the OBI image or DRR.

The optimizer's task is to find the global maximum of the similarity measure between the two images within the search space. The search space for the 2D‐2D automated image matching is the three rigid body degrees‐of‐freedom parameter‐set *P* (*x* translation, *y* translation, in plane rotation). The optimization is performed using a multiresolution approach that stepwise varies the image resolution and δ*P* at each step of the iterative process. The OBI software (version 1.0) provides two sets of multiresolution parameters based on trade‐offs with speed, precision, and robustness. One set uses larger steps and lower resolution of the images referred to as fast matching (FM), and the other set uses a higher resolution image sampling with smaller step sizes for precise matching (PM). The concept is to use the FM parameters for initial alignment and, if needed, use PM parameters for refining the final alignment. The default multiresolution parameters for both OBI automated image matching sets are provided in Table 1. The OBI and DRR images are 512×512 pixels with 12 bits per pixel (0−4095 gray scale range). The multiresolution parameters may be altered by the user at the time of image matching to steer the optimization process for automated image matching. Most of the measurements used the default parameters in the Table 1. If the automated image matching should fail, a backup procedure is to manually shift the DRR images until an acceptable registration is obtained. The manual matching is considered to be user‐dependent; the associated learning curve and quality aspects were previously analyzed.^(15)^

**Table 1:** Default parameters for OBI automated image matching

Steps	Fast matching		Precise matching	
	Resolution (pixels)	Step size (mm)	Resolution (pixels)	Step size (mm)
1	128	4	128	4
2	256	2	256	2
3	256	1	256	1
4	256	0.5	512	0.5

### C. Phantom verification procedure

**Figure 2:**
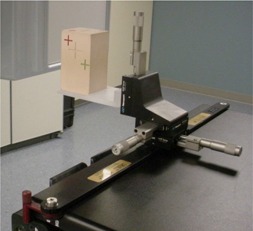
OBI geometric phantom consists of five small radio‐opaque markers $\left(2.5\times 1.0\;{\text{mm}}^{2}\right)$ embedded in lung‐equivalent material, and it is mounted on a Varian Exact™ Lok‐Bar with a cam‐lock locking mechanism incorporating a micrometer positioning tool for precisely moving the phantom in the couch lateral, longitudinal, and vertical directions.

Verification tests were performed on both a geometric rigid phantom and an anthropomorphic head phantom containing a humanoid skeleton to assess the precision and accuracy of the automated positioning system. The OBI geometric phantom (Varian Medical Systems, Palo Alto, CA) consists of five small radio‐opaque markers $\left(2.5\times 1.0\;{\text{mm}}^{2}\right)$ embedded in lung‐equivalent material. The geometric phantom, shown in Figure 2, is mounted on a Varian Exact™ Lok‐Bar (MedTec, Orange City, IA) with a cam‐lock locking mechanism incorporating a micrometer positioning tool for precisely moving the phantom in the couch lateral, longitudinal, and vertical directions. These movements performed with the micrometers are independent of the couch movement. The geometric phantom was scanned in a CT system (Lightspeed CT Scanner, GE Medical Systems, Milwaukee, WI) with a slice spacing of 2.5 mm and pixel resolution of 0.977 mm.

An anthropomorphic phantom (Radionics, Burlington, MA) was used to test the OBI system's performance for detection and correction of translational setup errors with and without rotational errors in the head. The head phantom consists of two pieces that are assembled together and eliminates problems of image matching with a segmented phantom. The phantom evaluates the entire procedure from CT‐scanning to treatment and verifying the alignment of treatment beam and target using a phantom similar to patient anatomy. Two CT datasets of the phantom were acquired with different slice spacing of 0.625 mm and 2.5 mm. The reason for the two CT data acquisitions was to investigate the possible influence of slice thickness in reconstruction of the DRR. In both scans each reconstructed slice image was 512×512 pixels at 0.428 mm pixel resolution. The image datasets were transferred to the Eclipse Treatment Planning System (Varian Medical Systems, Helsinki, Finland) to create DRRs, which were then transferred to an information system database (VARiS, Varian Medical Systems, Palo Alto, CA) for use with the OBI positioning system on the treatment machine.

The positional accuracy of the OBI kV X‐ray positioning system was compared against the micrometer positioning stage for the OBI geometric phantom and couch movements for the anthropomorphic phantom. The measurements in this investigation were repeated three to five times; averages and standard deviations were recorded in millimeters. Tabulated deviations are the differences between the actual position introduced by either the micrometer positioning stage for geometric phantom or couch positioning system for anthropomorphic phantom and the calculated position by the automated image registration algorithm for a particular co‐ordinate. The tests were repeated for both the PM and FM multistep matching parameters described in Table 1.

The coordinate system is defined as follows, with the patient in a supine position on the treatment couch, head to the gantry: vertical (being in the direction of the patient's posterior‐to‐anterior), longitudinal (being in the direction of the patient's superior‐to‐inferior), and lateral (being in the direction of the patient's right‐to‐left). Yaw rotation (couch angle rotation) is defined about the axis through the treatment isocenter and couch (patient's anterior‐posterior axis). Verellen and colleagues^(16)^ reported on a testing procedure for detection and correction of translational setup errors with and without rotational errors using a segmented phantom. The verification tests based on Verellen's method have been modified for analyzing the OBI automated positioning performance. The first tests were separate translations in the lateral, longitudinal, and vertical directions. The phantom offset varied depending on the geometric phantom or anthropomorphic phantom. The phantom translations and rotations were generated in both positive and negative directions. The geometric phantom translation offsets were 3 mm, 5 mm, and 9 mm. The anthropomorphic phantom translation offsets were 5 mm, 10 mm, and 15 mm. Tests were also performed with combined shifts in the three principal directions (positive or negative) with the phantom offset by 5 mm to 10 mm simultaneously. A third set of tests was performed for couch rotations (absence of translational shifts) of 2° and 5°. The final tests performed were combined translations and rotations of the phantoms, which would simulate a more realistic situation in the clinical use of OBI.

## III. RESULTS

Table 2 contains the overall mean magnitude of deviations for the geometric phantom verification of the automated image registration software. The shifts (generated in both positive and negative directions) did not show any directional sensitivity and were close to zero for the majority of the measurements. In this paper, we report the mean magnitude of the deviation, which is the average of the displacement error for each experimental setup regardless of the direction sign (±) of the shift. From the translation only studies, the average magnitude between the detected and known offset was less than 0.75 mm for each of the three principal directions. The results are given as the measurement with standard deviation in parentheses. The combined translations and rotations had the greatest average deviations in the lateral, longitudinal, and vertical directions, which were 1.5 (0.7), 0.5 (0.7), and 1.0 (0.0), respectively. Measurements recorded in Tables 3 and 4 for the rigid head phantom show the mean magnitudes of deviations between the detected and known positional offset for the 0.625 mm and 2.5 mm slice spacing, respectively. The maximal average deviation for the translation data was 0.9 mm for both CT datasets. As observed with the geometric phantom, the greatest average deviations were seen with the combined translation and rotational shifts. For the 0.625‐mm dataset, the lateral, longitudinal, and vertical average deviations for combined translations and rotations were 0.58 (0.5), 1.2 (0.4), and 1.0 (0), respectively. For the combined shifts and rotation, the average deviation between the known and detected offsets was 0.42 (0.51), 1.1 (0.38), and 0.75 (0.87) using the 2.5‐mm slice spacing CT dataset for the lateral, longitudinal, and vertical coordinates, respectively. Using these data points, the 3D offset vector was 1.4 mm. For small rotations of the head phantom, the average deviation between the detected and known rotational offset was 0.38° (0.3) and 0.30° (0.25) for the 0.625 mm and 2.5 mm slice spacing datasets. For dimensions in which translation was not actually introduced (rotation only), the detected average translation was below 1 mm among individual measurements and below 0.58 mm for average deviations.

**Table 2:** Overall mean displacement magnitudes (SD) for geometric phantom verification tests

Type	Couch lat (mm)	Couch long (mm)	Couch vert (mm)	Couch rtn (°)
translations only	0.63 (0.50)	0.31 (0.48)	0.73 (0.45)	0.51 (0.50)
combined translations	1.0 (0.89)	0.17 (0.41)	0.67 (0.52)	0.33 (0.26)
rotations only	0.25 (0.50)	0.0 (0.0)	0.50 (0.58)	0.43 (0.32)
combined translations and rotations	1.5 (0.71)	0.5 (0.71)	1.0 (0.0)	1.15 (1.2)

**Table 3:** Overall mean displacement magnitudes (SD) for anthropomorphic head phantom verification tests using 0.625‐mm slice spacing

Type	Couch lat (mm)	Couch long (mm)	Couch vert (mm)	Couch rtn (°)
translations only	0.11 (0.33)	0.44 (0.53)	0.0 (0.0)	0.47 (0.40)
combined translations	0.67 (0.51)	0.89 (0.41)	0.0 (0.0)	0.69 (0.32)
rotations only	0.50 (0.52)	0.58 (0.67)	0.50 (0.52)	0.38 (0.27)
combined translations and rotations	0.58 (0.51)	1.2 (0.38)	1.0 (0.0)	0.83 (0.32)

**Table 4:** Overall mean displacement magnitudes (SD) for anthropomorphic head phantom verification tests using 2.5‐mm slice spacing

Type	Couch lat (mm)	Couch long (mm)	Couch vert (mm)	Couch rtn (°)
translations only	0.11 (0.33)	0.33 (0.49)	0.50 (0.51)	0.26 (0.31)
combined translations	0.67 (0.49)	0.44(0.51)	0.89 (0.32)	0.56 (0.37)
rotations only	0.25 (0.45)	0.67 (0.49)	0.50 (0.52)	0.57 (0.40)
combined translations and rotations	0.42 (0.51)	1.1 (0.38)	0.75 (0.87)	0.30 (0.25)

## IV. DISCUSSION

This paper did not examine the geometric calibration and isocenter stability of the OBI system. Previous work by Zinniker and colleagues reported on the isocenter stability of a LINAC with OBI arms extended, which created a smaller effective isocenter.^(17)^ The authors explained that the added weight of OBI added a pulling force in the opposite direction to the forces normally produced by the weight of the LINAC components. The uncertainty of the kV system's isocenter location was reported to be much less than 1 mm.^(17)^

In our study, there were no significant differences between the PM and FM multistep matching parameters when using the automatch software. This is so because the four‐step process is identical except for the final step, when the full 512×512 pixel resolution is used for the PM parameters. The increased resolution for this final step did not result in any statistical differences between the PM and FM matching parameters’ results. For all dimensions, the magnitude of the deviation does not appear to be correlated with the magnitude of the actual translation introduced. We found no differences between the 0.625 mm and 2.5 mm acquisition data, which indicate the minimal influence of slice spacing on DRR reconstruction. However, Murphy reported the influence of CT slice thickness in radiographic patient positioning for 3.0‐mm and 1.5‐mm spacing.^(18)^The use of slice spacing greater than 2.5 mm may lead to poor DRR reconstruction, but that was not evaluated in this study. Our clinical institution uses 2.5‐mm slice spacing for all DRR reconstructions for patient treatment planning. Using the 2.5‐mm slice spacing, the average 3D offset vector was reported as 1.4 mm for the combined translations and rotation tests. The accuracy of the OBI system to detect a known offset using phantoms can be put into perspective by a few points. If using the 1.4‐mm 3D offset as the uncertainty for detecting a known offset, the pixel sizes of CT datasets may be 0.97 mm for typical patient scans, which is similar in scale. In addition, the dose calculation grid size used for treatment planning is greater than 2 mm for most treatment‐planning situations. Another point is that the graphical user interface for the OBI software reports the couch shifts in millimeter resolution, which prevents any possibility of submillimeter accuracy. Thus, an overall uncertainty in the positioning of known offsets is approximately 1.4 mm for most clinical cases. This type of uncertainty should be considered for target delineation when using image‐guided radiation therapy for consideration of margin reductions.

## V. CONCLUSION

The OBI system has been successfully integrated into a feasible online radiotherapy treatment‐guidance procedure. Use of an automated image registration system based on mutual information provides the clinician with an objective method of online patient repositioning. In the phantom verification tests performed in this study, the registration algorithm is capable of detecting known translations and rotations with an accuracy of less than 1.4 mm for a 3D vector offset (0.4 mm, 1.1 mm, and 0.8 mm in lateral, longitudinal, and vertical dimensions, respectively). This approach validates the use of this algorithm for online patient repositioning, but evaluation of each patient's resulting automatch should be performed by therapists before each treatment session for adequate clinical oversight.

## Supporting information

Supplementary Material Files
