# Supplementary figures and images for: Performance evaluation of an automated image registration algorithm using an integrated kilovoltage imaging and guidance system
Source: J Appl Clin Med Phys. 2006 Feb 21;7(1):97–104. doi: 10.1120/jacmp.v7i1.2199 (PMC5722475; doi:10.1120/jacmp.v7i1.2199)

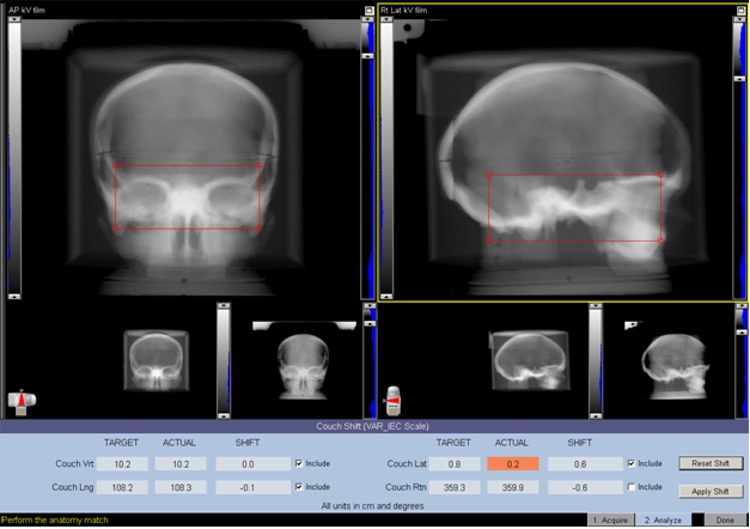

Supplement: Supplementary file 1 — Supplementary Material Files [file ACM2-7-097-s001.jpg]
